# Supplementary material for: Patterns of Intron Gain and Loss in Fungi
Source: PLoS Biol. 2004 Nov 30;2(12):e422. doi: 10.1371/journal.pbio.0020422 (PMC532390; doi:10.1371/journal.pbio.0020422)
Supplement: Table S1 — Also available at http://genes.mit.edu/NielsenEtAl/. (4.3 MB ZIP). [file pbio.0020422.st001.zip › NielsenEtAl/html/437.html]

AN1535.1.NCU03054.1.MG04759.1.FG00514.1


```
 CLUSTAL W (1.82) Multiple Sequence Alignments - Introns Inserted


Sequence 1: NCU03054.1	406 aa
Sequence 2: MG04759.1	412 aa
Sequence 3: AN1535.1	391 aa
Sequence 4: FG00514.1	427 aa
Alignment Length: 498 aa
Number Identitical Residues: 134 aa
Alignment Score (without introns) 6448


MG04759.1 	MGLRDLLRKKDGIEADGPGANEQTVD-QFGAQQQGHLRQPVTFIRTDTFNHEVIQPPGDP
NCU03054.1	MGL--FAKKKHGHEKGDSEAHSPSPNTQLGAP-------EITFVRTDTFTEERIFPPASP
FG00514.1 	MGIRDILKKKDDLSTGQP-AQNETVD-RLAAP-------EFTIIRSDTSTQERIYPPS--
AN1535.1  	MPLRDILHKKDRLNNTSDQYATDTLDAANSVP-------EIRFIRSDTTTQEIIIPPN--
          	* : .: :**.  .         : ::  ..         . ::*:** ..* * **   

MG04759.1 	AAEQDGGRDKGNSMHGGDGNFLSPDGSKRRSFFSSGSSRSRGSSESSAHSPTADPGHKDK
NCU03054.1	VTENDADHDSYLSAETASG--TPARGRLSLDVFRSNRSRSQSMS-SNPKSPGAKR-----
FG00514.1 	---MKSVDNLYLAAQDPKG---KEKPRRSLDVFRSGRSRSGSESSQTKKESGARR-----
AN1535.1  	---HDVDHEDRLASPRPSS---SSSHRRTLNPFHRSRTPPDPQD---PGSPRERR-----
          	    .   :   :    ..           . *  . : .   .     ..         

MG04759.1 	DKVFRRLSQRLHLARSPPSSENVPQNLPEI--VIRPE-EEASPAVESKWEERATILAMEN
NCU03054.1	-------ASRFHLHRSPVSSDNVPQDLPDVPAIVVPEGDEDKEGKEAQWEKRATMLARKN
FG00514.1 	------LSHRLHLSRAPPSSENVPENLPDIV-------TSSDGPDELQWEKRATMLAGQN
AN1535.1  	-------FSGFLRRGSSSSSVNLPPDLPQI-----AADAGDEQEREAQWEKRATVLAQQS
          	          :    :. ** *:* :**::     . .   .   * :**:***:** :.

MG04759.1 	ERVRSRPPSPPGGGGGSSGDVSGGFAHMSIGD----------HAGPSGRKRSPS---AVS
NCU03054.1	SEVRSRPGARP---ASPTRSVSNDFARMRLGSGPTEAEEWPLKSEPGAVTPAPSATKAVS
FG00514.1 	NRVRT--ASRP--------------------------------GTPSDSGRSEG--REVS
AN1535.1  	PRYGS-PSHSP----APS---------MGLGSE---------NVRPRSRSSSHS---RIN
          	 .  : .   *    ..:            ..             *     : .    :.

MG04759.1 	SKALD~DNIQEAIRLHEAGE~LEQSTKLFGKLADPEGDNNPLSQVLYGLALR~HGWGCQP
NCU03054.1	SKAID~DDIQQAIKWHEEGN~LEESTALFGKLADPEGSNNPLSQVLYGLALR2HGWGCTP
FG00514.1 	LQKID~EDIQEAIRLHEAGN~LELSTRMFGRLADPQGANNPLSQVLYGLALR2HGWGCAP
AN1535.1  	DPQGD0VNILEAIRLHEAGE1LEQSTSMFAQLADTTGANNALSQVLYGLALR2HGWGCRP
          	    *  :* :**: ** *: ** ** :*.:***. * **.*********** ***** *

MG04759.1 	DPEGAVHYLSKAASNAATVETLALQAGMKKGGAAKGELVLAIFELANCFRHGWGVQKDPV
NCU03054.1	DAAKAVTYLSAAASNAAEIEQLALQAGLKKGGAAKGELVLAIFELANCFRHGWGIPKDPI
FG00514.1 	DPEGAVKYLSAAASNSASIEQMALQAGMTKGGAAKGELVLAIFELANCFRHGWGINKDAY
AN1535.1  	DPEKAVVYLSAAASNSASIEAQALEAGLKKGGAAKGELVLAIFELGNCFRNGWGVKKDPA
          	*.  ** *** ****:* :*  **:**:.****************.****:***: **. 

MG04759.1 	AAKQ0YYETAANLGDTD1AMNEVAWCYLEGFGCKKDK0--FASAKYYRLAEK-------~
NCU03054.1	AAKQ~YYETAANLGDSD1AMNEVAWCYLEGFGTKKDK0--YTAAKYYRLAEK-------~
FG00514.1 	AAKQ0YYETAANLGDTD1AMNEIAWCYVEGFGCKKDK0SKYTSQRVLGPTEALIPHIQI1
AN1535.1  	AARQ~YFETAANLGDTD~AMNEVAWCYLEGFGGKKDK0--FMAAKYYRLAES-------~
          	**:* *:********:* ****:****:**** ****   : : :    :*         

MG04759.1 	SGNKTLGNSW2IWKEKYD---P~GNNGKKK----------------~-------------
NCU03054.1	NGNKIMGNTC2HFPRKYQ---V~EKGGLMPGRLR------------~-------------
FG00514.1 	RSSPVLSTGR~EVRQQDTRELL0DLEGKVRPSGRGQPQVVRRRQPF1NFVEEEEDATLRW
AN1535.1  	KGNKIVGNSC2KSAHLQP---L~SKTGTRHLGAPAQP--YHAPQPY~HAPGSRTDG----
          	 ..  :..      .           *       ...      ..      .  ..    

MG04759.1 	----------------~------------
NCU03054.1	----------------~------------
FG00514.1 	AAGKGPGLSVGDYIVF1LDVCCPSPNTTG
AN1535.1  	----------------~------------
          	
```
